# Phosphorylation of Golgi Peripheral Membrane Protein Grasp65 Is an Integral Step in the Formation of the Human Cytomegalovirus Cytoplasmic Assembly Compartment

**DOI:** 10.1128/mBio.01554-16

**Published:** 2016-10-04

**Authors:** G. Michael Rebmann, Robert Grabski, Veronica Sanchez, William J. Britt

**Affiliations:** a Department of Microbiology, University of Alabama School of Medicine, University of Alabama in Birmingham, Birmingham, Alabama, USA; b Department of Pediatrics, University of Alabama School of Medicine, University of Alabama in Birmingham, Birmingham, Alabama, USA; c Department of Neurobiology, University of Alabama School of Medicine, University of Alabama in Birmingham, Birmingham, Alabama, USA

## Abstract

Human cytomegalovirus (HCMV) is the largest member of the Herpesviridae and represents a significant cause of disease. During virus replication, HCMV alters cellular functions to facilitate its replication, including significant reorganization of the secretory and endocytic pathways of the infected cell. A defining morphologic change of the infected cell is the formation of a membranous structure in the cytoplasm that is designated the virion assembly compartment (AC), which consists of virion structural proteins surrounded by cellular membranes. The loss of normal Golgi compartment morphology and its relocalization from a juxtanuclear ribbonlike structure to a series of concentric rings on the periphery of the AC represents a readily recognized reorganization of cellular membranes in the HCMV-infected cell. Although trafficking of viral proteins to this compartment is required for the assembly of infectious virions, the functional significance of the reorganization of intracellular membranes like the Golgi membranes into the AC in the assembly of infectious virus remains understudied. In this study, we determined that Golgi membrane ribbon fragmentation increased during the early cytoplasmic phase of virion assembly and that Golgi membrane fragmentation in infected cells was dependent on the phosphorylation of an integral *cis-*Golgi protein, Grasp65. Inhibition of Golgi membrane fragmentation and of its reorganization into the AC resulted in decreased production of infectious particles and alteration of the incorporation of an essential protein into the envelope of the mature virion. These results demonstrated the complexity of the virus-host cell interactions required for efficient assembly of this large DNA virus.

## IMPORTANCE

The human cytomegalovirus (HCMV)-induced reorganization of intracellular membranes that is required for the formation of the viral assembly compartment (AC) has been an area of study over the last 20 years. The significance of this virus-induced structure has been evinced by the results of several studies which showed that relocalization of viral proteins to the AC was required for efficient assembly of infectious virus. In this study, we have identified a mechanism for the fragmentation of the Golgi ribbon in the infected cell *en route* to AC morphogenesis. Identification of this fundamental process during HCMV replication allowed us to propose that the functional role of Golgi membrane reorganization during HCMV infection was the concentration of viral structural proteins and subviral structures into a single intracellular compartment in order to facilitate efficient protein-protein interactions and the virion protein trafficking required for the assembly of this large and structurally complex virus.

## INTRODUCTION

Human cytomegalovirus (HCMV) is a ubiquitous human pathogen that is estimated to infect between 50 and 80% of the adult population in the United States and an even higher proportion of populations in lower-income countries (1). In normal individuals, HCMV is infrequently associated with clinical symptoms, and yet, it remains a significant cause of mortality and morbidity in immunocompromised individuals, such as patients receiving immunosuppressive drugs (1). Intrauterine HCMV infection of the developing fetus has been shown to result in abnormal brain development that leads to long-term neurological sequelae, including hearing loss in 10% of infants infected *in utero* (2, 3).

Infection of human dermal fibroblast cells (HF) with laboratory strains of HCMV has been used to study lytic infection, including the interactions between viral and cellular proteins that lead to the assembly of infectious virus particles. To accommodate an extended eclipse period and the assembly of a structurally complex virion, HCMV utilizes multiple strategies to regulate the intracellular environment for its replication. These mechanisms include (i) inhibiting innate defense mechanisms, (ii) blocking the activation of both the extrinsic and intrinsic cellular apoptotic signaling pathways, (iii) inhibiting endoplasmic reticulum (ER) stress responses and autophagy, and (iv) dysregulating cell cycle signaling pathways (4–16). In addition, HCMV infection has been shown to result in increased activation of the mitotic kinase Cdk1 (15, 17). Although the importance of mitotic kinase activity in the replication of HCMV remains to be fully defined, previous studies using the pan-CDK inhibitor roscovitine demonstrated a dose-dependent decrease in infectious virus production (13).

Similar to the assembly of other herpesviruses, the assembly of HCMV progeny virions is a complex process involving both a nuclear and cytoplasmic phase. Subviral particles acquire the tegument proteins and the lipid envelope containing virus-encoded glycoproteins (secondary envelopment) within a stable, virus-induced membranous structure that was initially designated the assembly compartment (AC) and subsequently termed the cytoplasmic virus assembly compartment (18–20). The AC is a morphologically defined structure consisting of reorganized membranes of the secretory and endocytic systems of the cell, as well as virion tegument and envelope proteins (18, 19, 21, 22). The AC is located in a juxtanuclear position and overlaps the microtubule-organizing center (MTOC) (18).

Although the mechanisms leading to the morphogenesis of the AC remain to be fully elucidated, the accumulation of viral tegument proteins, glycoproteins, and enveloped virus particles within the AC suggests that the formation of this specialized structure is essential for the process of secondary envelopment (18, 20). The dependence of viral assembly upon AC morphogenesis is further supported by studies that have shown that interference with trafficking of viral proteins to this structure resulted in decreased production of infectious virions (18, 22–30). Similarly, mutation and deletion of viral microRNAs (miRNAs) targeting components of the secretory and endocytic pathways has been shown to disrupt the AC and dramatically inhibit the production of infectious virus (31). Thus, the major reorganization of the secretory compartment (endoplasmic reticulum-Golgi intermediate compartment [ERGIC], Golgi compartment, and the endolysosomal network) in HCMV-infected cells that results in the formation of the AC is linked to the assembly of infectious virus, and yet, the pathways leading to reorganization of intracellular membranes in virus-infected cells have not been characterized.

One of the most dramatic changes in the distribution of intracellular membranes in HCMV-infected cells is the reorganization of the Golgi apparatus into a concentric ring that defines the outer border of the AC (18, 19, 32). Throughout interphase, the Golgi apparatus is organized as a series of stacks connected by tubule extensions to create a homogeneous organelle, the Golgi ribbon, which allows diffusion of enzymes within specific cisternae (33). The Golgi membrane reassembly stacking proteins (Grasp), Grasp65 and Grasp55, are multifunctional peripheral membrane proteins that are localized to the *cis* and medial cisternae, respectively, of the Golgi membranes and that contribute to the structural integrity of the Golgi membrane stacks (34–37). Homo-oligomerization between Grasp65 and Grasp55 monomers on adjacent cisternae helps to maintain the structural integrity of the Golgi membranes and is mediated by interactions between domains in the N termini of the Grasp proteins (38). Posttranslational modification of the Grasp proteins has been shown to be essential for the disassembly of the Golgi membranes during apoptosis or mitosis (36, 39–43). During mitosis, both Grasp proteins are phosphorylated at multiple residues through a well-characterized process that results in the fragmentation of the Golgi membrane ribbon and subsequent segregation of organelle membrane fragments into nascent daughter cells (34, 41, 44, 45). Mutations of consensus sequences within Grasp65 and Grasp55 that are proposed targets of mitotic kinases have been shown to delay Golgi membrane stack disassembly during cell division (37, 41, 43).

In this study, we defined the kinetics of Golgi membrane disassembly and AC formation during HCMV infection. We demonstrated that Grasp65 phosphorylation provides a mechanism for the disassembly of the Golgi apparatus during HCMV infection, prior to the formation of the AC. The expression of a Grasp65 protein containing mutations at mitotic phosphorylation sites delayed Golgi membrane fragmentation, AC formation, and the assembly of infectious virus, possibly by altering the efficiency of the incorporation of essential viral glycoproteins into the virion. Together, our data indicated that fragmentation and redistribution of the *cis-*Golgi membranes into the outer boundary of the AC was required for the efficient production of infectious virus particles.

## RESULTS

### The Golgi membrane stacks are modified early in infection and disassembly of the Golgi apparatus is linked temporally with the cytoplasmic expression of the viral tegument protein pp65.

**FIG 1:**
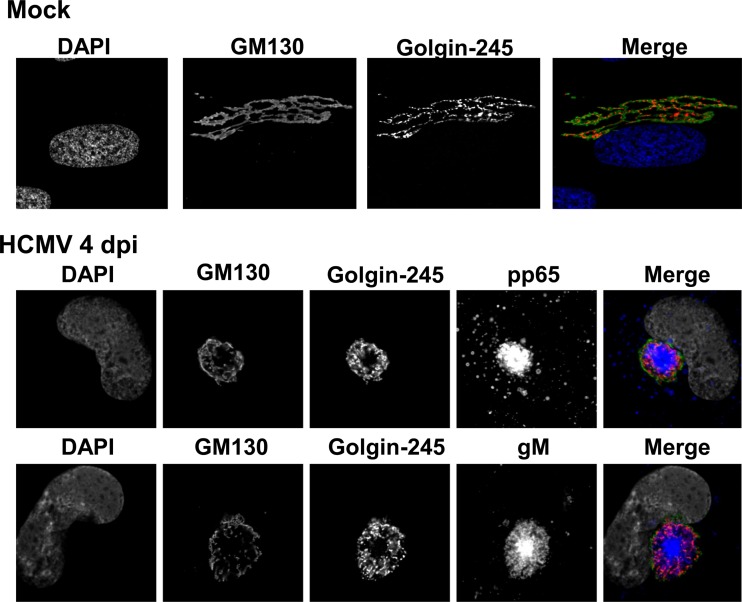
Golgi apparatus-derived membranes form the outer boundary of the HCMV cytoplasmic assembly compartment. HF cells were plated on glass coverslips and allowed to grow to confluence, followed by a 3-day incubation in serum-free medium. Cells were then infected at an MOI of 1. Coverslips were collected at 4 days p.i. and analyzed as described in Materials and Methods. Top, mock-infected cells were stained with antibodies reactive with *cis-*Golgi marker GM130 (merge: green), *trans*-Golgi marker golgin-245 (merge: red), and DAPI (merge: blue). Middle, HCMV AD 169-infected cells were stained with antibodies directed against GM130 (merge: green), golgin-245 (merge: red), pp65 (merge: blue), and DAPI (merge: gray). Bottom, HCMV AD 169-infected cells were stained with antibodies reactive with GM130 (merge: green), golgin-245 (merge: red), gM (merge: blue), and DAPI (merge: gray). Representative images are shown.

**FIG 2:**
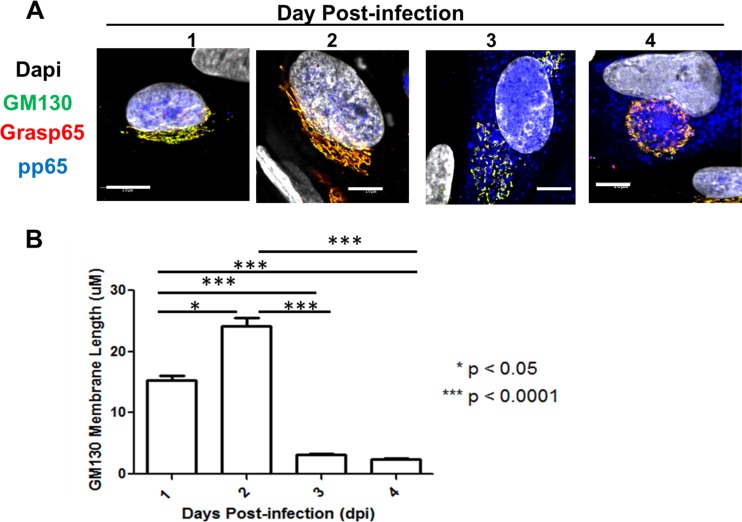
Kinetics of Golgi membrane fragmentation during HCMV infection. HF cells were plated on glass coverslips and infected as described in the legend to Fig. 1. (A) Coverslips were collected on days 1 to 4 p.i. and stained with antibodies reactive with GM130 (green), Grasp65 (red), pp65 (blue), and DAPI (gray). Representative images demonstrate intact Golgi membrane ribbon (days 1 and 2), fragmented Golgi membrane ribbons (day 3), and the AC (day 4). Scale bar is equal to 10 µm. (B) The length of *cis*-Golgi tubules is dynamically altered during HCMV infection. GM130^+^ membrane length on the indicated day postinfection was measured using the trace feature in FluoView software 10.1. Median values are plotted, and error bars show standard deviations. The significance of differences between groups was determined using the Kruskal-Wallis test (*, *P* < 0.05; ***, *P* < 0.001). The number of cells analyzed each day was as follows: day 1, *n* = 100; day 2, *n* = 120; day 3, *n* = 80; day 4, *n* = 75.

**FIG 3:**
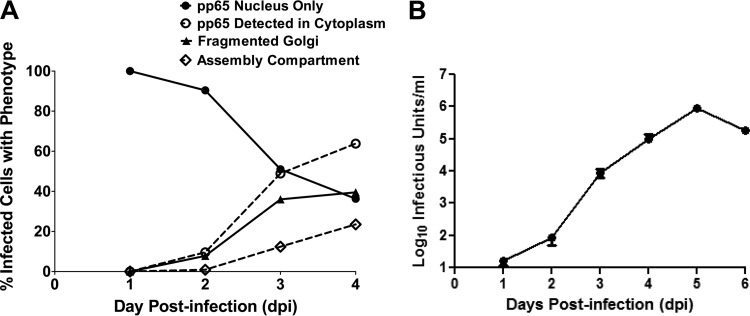
Fragmentation of the Golgi membrane during HCMV infection correlates temporally with pp65 cytoplasmic accumulation, AC morphogenesis, and virus production. Confocal microscopy was used to score the number of infected cells displaying the phenotypes illustrated in Fig. 2A. (A) Percentages of infected cells with fragmented Golgi membrane ribbon, presence of fully formed AC, nuclear pp65 localization, and cytoplasmic pp65 localization at the indicated time points. Percentages were calculated based on the total number of infected cells counted at each time point; the numbers of cells counted and percentages are listed in Table 1. (B) The detection of the cytoplasmic AC correlated with the logarithmic increase in virus production. Viral titers were determined as described in Materials and Methods. Data are representative of three independent experiments. The results from one experiment are shown. Error bars show standard deviations.

Our initial experiments utilized HCMV strain AD 169-infected human fibroblasts (HF), antibodies reactive with Golgi membrane-specific proteins, and monoclonal antibodies (MAbs) reactive with virion proteins to define the redistribution of Golgi apparatus-derived membranes late in infection (Fig. 1). The images in the middle and bottom panels of Fig. 1 illustrate the formation of a distinct juxtanuclear AC in which the Golgi membrane markers GM130 (*cis-*Golgi) and golgin-245 (*trans-*Golgi) were localized to the region surrounding an area in which virion protein pp65 (UL83) or gM (UL100) was concentrated. To investigate the modification of the Golgi membrane ribbon during HCMV infection, we tracked morphological changes in the Golgi membranes relative to the expression and intracellular localization of HCMV early and late proteins as markers of the stage of virus replication. Using confocal microscopy, we determined that the *cis*-Golgi ribbon (GM130-positive [GM130^+^] membranes) increased in transverse length by approximately 10 µm in infected cells between 1 and 2 days postinfection (p.i.) (Fig. 2A, days 1 and 2, and B). However, beginning on day 2 p.i., the Golgi apparatus was fragmented and dispersed in the cytoplasm in approximately 8% of infected cells, which were distinguished by the expression of the viral tegument protein pp65 (Table 1 and Fig. 3A). The frequency of infected cells containing fragmented Golgi membranes increased between 2 and 3 days p.i. to approximately 36% (Fig. 2A, day 3, and Table 1). During the same interval, the percentage of cells with an AC, defined as a juxtanuclear accumulation of virion proteins (pp65 or gM) surrounded by Golgi apparatus-derived membranes (Fig. 2A, day 4), increased from 0.9% to 12.4%. Interestingly, 90% and 98% of cells that expressed pp65 in the cytoplasm also contained a modified Golgi membrane structure (fragmented Golgi membranes or distinct AC) on days 2 and 3 p.i., respectively (Table 1 and Fig. 3A). The percentages of cells with fragmented Golgi membranes (~39%) or an AC (~24%) increased on day 4 p.i., a finding that also correlated with an increase in the percentage of cells in which pp65 was detected in the cytoplasm (Table 1 and Fig. 3A). This finding suggested that cytoplasmic expression of pp65 could be used as a surrogate marker for the shift in the viral assembly process from a nuclear phase to the cytoplasmic events of secondary envelopment and the assembly of infectious virions. At days 3 and 4 p.i., the average length of GM130^+^ membranes in infected cells decreased to 2 to 3 µm (Fig. 2B). Finally, the expression of the viral glycoprotein gM coincided with the coalescence of the individual GM130^+^ Golgi membrane fragments into a ring-like structure on the periphery of the AC (data not shown), a morphological change that also coincided with both the expression of pp65 in the cytoplasm of infected cells and the continued logarithmic increase in virus production on days 3 and 4 p.i. (Fig. 2A and 3B and Table 1). These findings demonstrated that the Golgi apparatus was dynamically altered throughout the course of HCMV infection and suggested that the fragmentation of the Golgi membrane ribbon was an initial step in the reorganization of the Golgi apparatus during the formation of the AC in the cytoplasmic phase of virion assembly.

**Table 1:** Kinetics of Golgi membrane fragmentation and AC formation relates to production of infectious virus particles

Day p.i. (no. of cells examined)	No. (%) of cells with indicated phenotype^a^					Amt of infectious virus produced (IU/ml)
	pp65 in:		Golgi membrane morphology		Assembly compartment	
	Nucleus only	Cytoplasm	Intact	Fragmented		
1 (108)	108 (100)	0	108 (100)	0	0	0
2 (219)	198 (90.4)	21 (9.6)	200 (91.3)	17 (7.7)	2 (0.91)	9.6 × 101
3 (338)	173 (51.2)	165 (48.8)	175 (51.8)	121 (35.8)	42 (12.4)	9.4 × 103
3 (400)	145 (36.3)	255 (63.7)	151 (37.8)	155 (38.7)	94 (23.5)	1.02 × 105

a HF cells were plated on coverslips and allowed to grow to confluence, after which they were serum starved for 3 days. Cells were then infected at an MOI of 1. Coverslips were collected every 24 h p.i. and probed with antibodies specific for the *cis*-Golgi proteins GM130 and Grasp65. The viral protein pp65 was detected using 28-19 antibody, and DAPI was used to identify the nucleus. Cells were scored for the presence of pp65 in the nucleus only or in the nucleus and cytoplasm, as well as for the morphology of the Golgi membranes (fragmented or intact) at each time point analyzed. Golgi membranes were identified as fragmented if the majority of GM130^+^ membranes had a disrupted staining pattern with spaces interrupting adjacent or proximal GM130^+^ membranes. Values listed are representative of three individual experiments.

### Immediate early gene expression is not sufficient to induce the fragmentation of the Golgi membrane ribbon.

**FIG 4:**
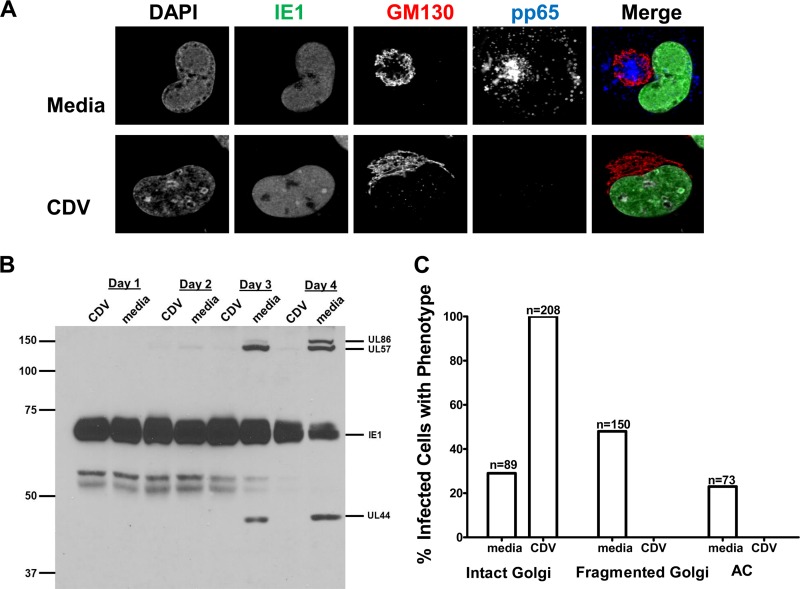
Treatment of HCMV-infected cells with the antiviral cidofovir inhibits Golgi membrane fragmentation and AC formation. HF cells were plated on glass coverslips and infected as described in the legend to Fig. 1. The inoculum was removed and replaced with medium or medium containing 30 µM cidofovir. Coverslips were fixed in PFA on day 4 p.i., and cells were analyzed for viral protein expression and changes in Golgi membrane morphology by confocal microscopy. (A) Intact Golgi membrane morphology was retained when HCMV-infected cells were treated with cidofovir. Cells were stained with antibodies reactive with IE1 (merge: green), pp65 (merge: blue), GM130 (merge: red), and DAPI (merge: gray). Top, medium-treated cells (media); bottom, cidofovir-treated cells (CDV). (B) Immunoblot of IE1 expression and early and late virus-encoded proteins in CDV-treated and untreated HCMV-infected cells. Cultures of infected cells were treated with 30 µM CDV or left untreated and harvested on the indicated days postinfection. Lysates were subjected to immunoblotting and probed with a mixture of anti-IE1 (exon 4), anti-UL57, anti-UL86 (MCP), and anti-UL44 MAbs. Note the lack of expression of early and late proteins in CDV-treated cells. Numbers to the left show molecular weight (in thousands). (C) Cidofovir treatment inhibits Golgi membrane fragmentation and AC morphogenesis. Confocal microscopy was used to score the numbers of infected cells displaying the phenotypes illustrated in panel A. The number of cells analyzed is listed above each bar. Cells with fragmented Golgi membranes or AC were not identified in the CDV-treated cultures. Experiments were repeated 2 times, and data shown are from one representative experiment.

To determine which class of viral genes was responsible for the fragmentation of Golgi membrane stacks, we infected confluent cells at a multiplicity of infection (MOI) of 1 and treated these cultures with the antiviral drug cidofovir (CDV) at a concentration of 30 µM from the onset of infection (*t* = 1) (Fig. 4). This dose of CDV has been shown to block both early and late HCMV gene expression but not the expression of the immediate early genes IE1, IE2, and UL36 (Fig. 4A and B) (M. Prichard, unpublished data). Similar percentages of cells expressing the immediate early 1 (IE1) protein, pp72, were detected in both treated and untreated cells (data not shown), and CDV blocked the expression of the early virus-encoded proteins pp65, UL57, and UL44 and the late protein UL86 (MCP) (Fig. 4A and B). At 4 days p.i., IE1-positive (IE1^+^) cells treated with CDV did not contain fragmented Golgi membrane ribbons and did not exhibit morphological changes associated with the formation of the AC (Fig. 4A and C). In untreated infected cells, the percentages of IE1^+^ cells with fragmented Golgi membranes or AC were 48% and 29%, respectively (Fig. 4C). These data demonstrated that the expression of immediate early genes, as evidenced by the expression of IE1, was not sufficient to induce Golgi membrane fragmentation, a finding that was consistent with the previous observations of the kinetics of Golgi membrane fragmentation and AC formation. Together these data suggested that early and/or late viral functions regulate this process by directly modifying cellular proteins responsible for the maintenance of Golgi membrane architecture or, alternatively, by indirectly regulating the expression of cellular functions as has been proposed in previous studies (46).

### Grasp65 phosphorylation during HCMV infection correlates with the fragmentation of the Golgi membrane ribbon.

**FIG 5:**
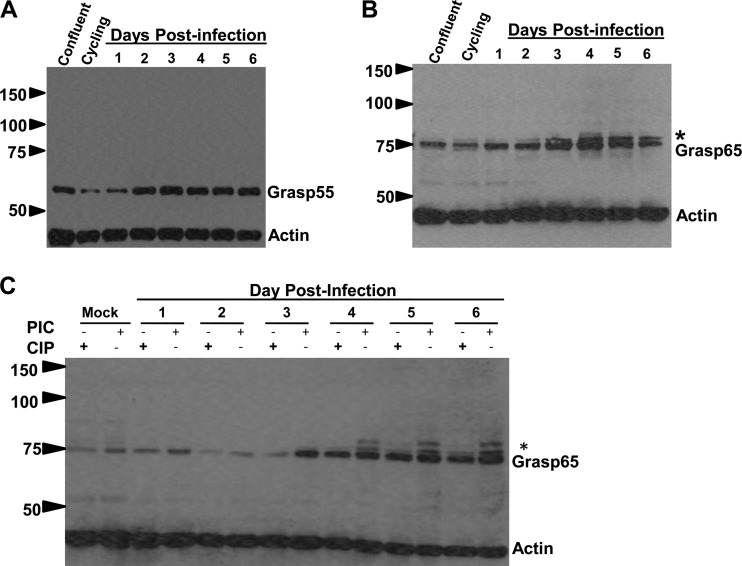
Grasp65 is phosphorylated during HCMV infection. HF cells were plated and grown to confluence prior to serum starvation for 72 h to synchronize cells. Cultures were infected with HCMV AD 169 at an MOI of 1. (A) Steady-state levels of Grasp55 are increased by HCMV infection compared to the level in an uninfected, asynchronous population (cycling) or uninfected, noncycling cells (confluent). (B) Grasp65 levels increase during infection, and higher-molecular-weight forms can be detected in cycling and infected cells. (C) HCMV infection induces higher-molecular-weight forms of Grasp65 that are eliminated with calf intestinal phosphatase (CIP) treatment but not by incubation in a cocktail of phosphatase inhibitors (PIC). Numbers to the left of each gel show molecular weight (in thousands).

Previous studies have shown that HCMV infection disrupted the cellular processes of apoptosis and autophagy, in addition to inducing the activity of the mitotic kinase Cdk1 (4, 7, 10, 12, 15, 16, 47). The activation of Cdk1 by HCMV infection could result in the fragmentation of the Golgi membrane ribbon via phosphorylation of Grasp proteins, as has been described during cell division (37, 41–43, 48). We next determined whether the Golgi membrane proteins Grasp65 and Grasp55 were posttranslationally modified during HCMV infection. To determine the effects of HCMV infection on the expression and posttranslational modifications of Grasp proteins in infected HF cells, cell lysates were collected at 24-h intervals from infected cells and analyzed by immunoblotting (Fig. 5A and B). As additional controls, lysates from a subconfluent, asynchronous population of proliferating cells and a confluent cell population were analyzed in parallel.

Utilizing Grasp-specific antibodies, we detected more slowly migrating forms of Grasp65 on days 2 to 6 p.i. (Fig. 5B). A similarly migrating higher-molecular-weight species, albeit in lesser amounts, was also observed in the sample from the uninfected proliferating cells but not from confluent cells (Fig. 5B). This form of Grasp65 may correspond to phosphorylated Grasp65 present during mitosis. In contrast, we did not detect changes in the migration of Grasp55 in the infected or uninfected samples (Fig. 5A). Interestingly, we also detected increases in Grasp65 and Grasp55 protein levels on days 3 to 6 p.i. relative to the levels in uninfected confluent control cells, findings consistent with the results from a proteomic analysis of HCMV-infected cells (Fig. 5A and B) (49). To determine whether the slower migration of Grasp65 was due to phosphorylation, infected-cell lysates were treated with calf intestinal phosphatase (CIP) or a mixture of phosphatase inhibitors (phosphatase inhibitor cocktail [PIC]). Treatment with CIP nearly eliminated the higher-molecular-weight forms of Grasp65 in infected samples, indicating that Grasp65 was modified by phosphorylation during HCMV infection (Fig. 5C). Together, these data demonstrated that the phosphorylation of Grasp65 correlated temporally with the fragmentation of the Golgi membrane stacks and AC formation and suggested that disassembly of the Golgi membrane ribbon during HCMV infection was linked to AC morphogenesis.

### Expression of a phosphorylation-negative Grasp65 mutant reduces Golgi membrane fragmentation and delays AC formation.

**FIG 6:**
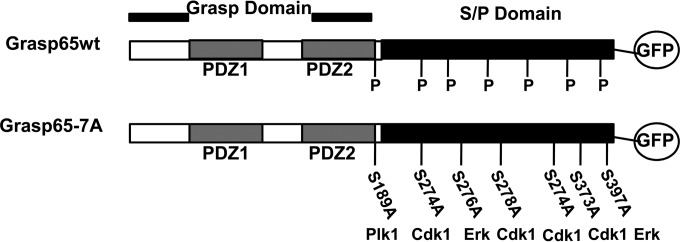
Construction of the Grasp65 phosphorylation-negative mutant. Grasp65wt was cloned into pEGFP-N1 to generate Grasp65-GFP. Overlapping mutagenic primers were used to generate alanine mutations at serines and threonines located in mitotic kinase target sequences in the S/P domain. The resulting mutant was designated Grasp65-7A. To generate lentiviral constructs, wt and mutant Grasp65-7A–GFP constructs were subcloned into the pLVX-puro vector.

**FIG 7:**
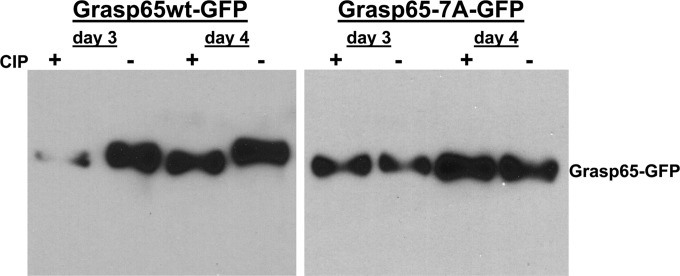
Grasp65-7A–GFP mutant is hypophosphorylated compared to Grasp65wt-GFP in HCMV-infected cells. HF cells were transduced with recombinant lentiviruses expressing either Grasp65wt-GFP or Grasp65-7A–GFP and synchronized prior to infection with HCMV AD 169 at an MOI of 1. Cultures were harvested on day 3 and 4 postinfection, and lysates analyzed for Grasp65wt or Grasp65-7A GFP-tagged protein as described in the legend to Fig. 5. Treatment of infected cell lysates with calf intestinal phosphatase (CIP +) increased the migration of higher-molecular-weight species of Grasp65wt-GFP but did not alter the migration of Grasp65-7A–GFP, suggesting that the Grasp65-7A–GFP mutant is hypophosphorylated during infection compared to Grasp65wt-GFP.

The kinetic correlation between Grasp65 phosphorylation, Golgi membrane fragmentation, and AC formation suggested that the virus regulated the activity of mitotic kinases to facilitate the cytoplasmic phase of the virion assembly process. To test this hypothesis, we mutated Grasp65 at 7 amino acids (S→A and T→A) corresponding to mitotic kinase consensus sequences, including the Cdk1/cyclin B, Plk1, and Erk sites, and named the mutant Grasp65-7A (Fig. 6) (37, 42, 48, 50). In the context of HCMV infection, these mutations in Grasp65 could be predicted to delay Golgi membrane fragmentation in a manner analogous to what has previously been described for mitotic cells (35, 37, 43, 51). The wild-type (wt) Grasp65 (Grasp65wt) and phosphorylation-negative Grasp65-7A mutant were first fused to enhanced green fluorescent protein (EGFP) at the C terminus of the Grasp65 coding sequence, and recombinant lentiviruses expressing the Grasp65wt-GFP or mutant Grasp65-7A–GFP protein were used to create stable cell lines. To determine whether the mutations placed within the Grasp65-7A protein sequence prevented its phosphorylation in the context of HCMV infection, stable cell lines expressing either the Grasp65wt-GFP or Grasp65-7A–GFP mutant protein were infected with HCMV at an MOI of 1, and cell lysates were assayed by immunoblotting using an anti-GFP antibody. As observed in HF cells, HCMV infection of the HF cell line expressing Grasp65wt-GFP led to the accumulation of a more slowly migrating form of Grasp65-GFP in lysates that were not treated with CIP, whereas phosphatase treatment of the infected-cell lysates with CIP increased the mobility of Grasp65wt-GFP, indicating that the protein was posttranslationally modified by phosphorylation (Fig. 7). In contrast, the migration of the Grasp65-7A–GFP mutant protein from HCMV-infected cells was not altered by treatment with CIP, suggesting that it was not posttranslationally modified to the same extent as Grasp65wt-GFP (Fig. 7).

**FIG 8:**
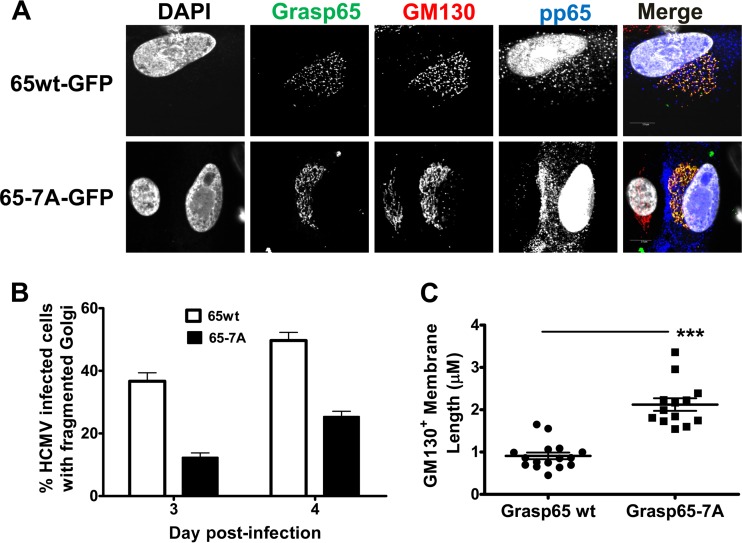
Expression of Grasp65-7A–GFP reduces fragmentation of the Golgi membrane ribbon in HCMV-infected cells. HF cells were transduced with recombinant lentiviruses expressing either Grasp65wt-GFP or Grasp65-7A–GFP, plated on coverslips, and synchronized prior to infection with HCMV AD 169 at an MOI of 1. Coverslips were collected on days 3 and 4 p.i. and then stained with antibodies to detect GM130 (merge: red), pp65 (merge: blue), and DAPI (merge: gray). (A) The phosphorylation-negative Grasp65-7A mutant inhibits Golgi membrane fragmentation. Panels show representative images of Golgi membrane morphology in cells expressing Grasp65wt-GFP (top) or Grasp65-7A–GFP (bottom) at day 3 p.i. (B) Confocal microscopy was used to score the number of infected cells displaying fragmented Golgi membrane on days 3 and 4 postinfection. Percentages were calculated based on the total number of infected cells (Grasp65wt, *n* = 225; Grasp65-7A, *n* = 238) counted at each time point. The experiment was repeated 2 times, and the data shown are from a single experiment. Error bars show standard deviations. (C) GM130^+^ membrane tubules are longer in cells expressing Grasp65-7A–GFP than in cells expressing Grasp65wt-GFP. The Freehand Trace feature in the FluoView 10.1 software (Olympus Corporation) was used to measure the length of individual GM130^+^ membrane tubules in a subset of infected cells displaying the fragmented Golgi membrane phenotype (Grasp65wt-GFP, *n* = 16; Grasp65-7A–GFP, *n* = 13). Individual membrane lengths are plotted, and median values and standard deviations are shown. Differences between groups were analyzed using the Mann-Whitney test (*, *P* < 0.05; ***, *P* < 0.001). The experiments were repeated twice, and the data shown are from one experiment.

We next infected Grasp65wt- or Grasp65-7A-expressing cells with HCMV to determine the effects of infection on Golgi membrane morphology and virus production. On day 3 post-HCMV infection, the expression of the Grasp65-7A mutant reduced the percentage of HCMV-infected cells with the fragmented Golgi membrane phenotype by approximately 50% compared to the number of cells expressing Grasp65wt (Fig. 8A and B). The incomplete effect of the Grasp65-7A mutant on the Golgi membrane fragmentation phenotype, defined as points of separation between distinct GM130^+^ membrane tubules, was likely secondary to the continued expression of endogenous Grasp65. It is important to note that the retention of Golgi membrane ribbon morphology resulting from the expression of Grasp65-7A did not affect the kinetics of pp65 expression. Thus, the effect of the Grasp65-7A mutant on the phenotype of the Golgi membranes was specific and did not appear to be a nonspecific effect on the viral replication program.

**FIG 9:**
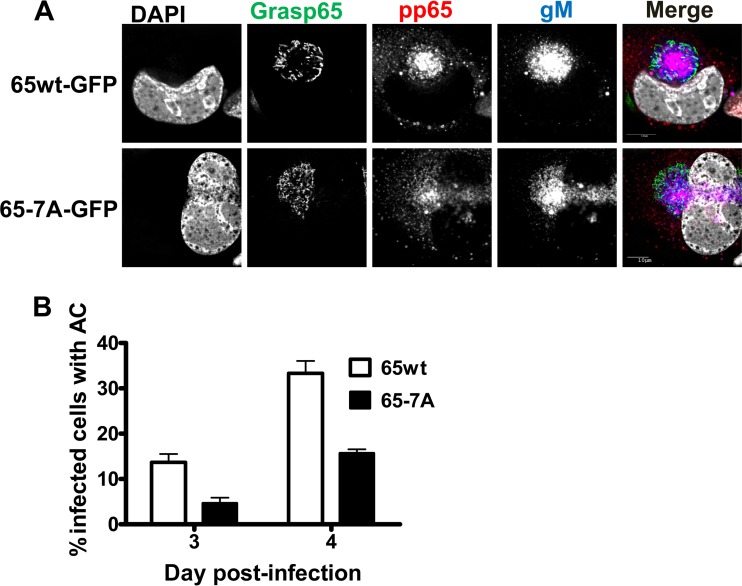
Expression of the Grasp65-7A–GFP mutant inhibits AC morphogenesis. HF cell lines expressing either Grasp65wt-GFP or Grasp65-7A–GFP were plated on glass coverslips and allowed to grow until confluent prior to infection with HCMV AD 169 at an MOI of 1. Coverslips were collected 3 and 4 days p.i. and fixed with PFA. Coverslips were stained with antibodies to viral proteins pp65 (merge: blue), the envelope protein gM (merge: red), and DAPI (merge: gray). (A) The phospho-negative Grasp65-7A mutant inhibits AC formation. Panels show representative images of the AC in cells expressing Grasp65wt-GFP (top) or Grasp65-7A–GFP (bottom) at day 4 p.i. (B) Confocal microscopy was used to score the number of GFP-positive, HCMV-infected cells displaying a fully formed AC as shown in panel A. Percentages were calculated based on the total numbers of infected cells (Grasp65wt, *n* = 205; Grasp65-7A, *n* = 219) counted at each time point. The experiment was repeated 2 times, and the data shown are from a single experiment. Error bars show standard deviations.

To further quantify the effects of Grasp phosphorylation on Golgi membrane structure in the context of HCMV infection, we measured the length of individual GM130^+^ membrane tubules in cells expressing either Grasp65wt or Grasp65-7A and displaying the fragmented Golgi membrane phenotype. The GM130^+^ membrane tubules were approximately twice as long in cells expressing the Grasp65-7A mutant as in those expressing Grasp65wt (Fig. 8C). In addition, relative to the results for Grasp65wt-expressing cells, Grasp65-7A expression led to a 50% reduction of infected cells with an AC, which was defined as a concentrated area of gM expression surrounded by Golgi apparatus-derived membranes (Fig. 9A and B). These data supported our hypothesis that phosphorylation of Grasp65 during HCMV infection led to fragmentation of the Golgi membranes and that this was required for AC formation. Finally, these data suggested a critical role for mitotic kinase activity during the cytoplasmic phase of HCMV virion assembly.

### Expression of Grasp65-7A reduces the production of infectious virus particles.

**FIG 10:**
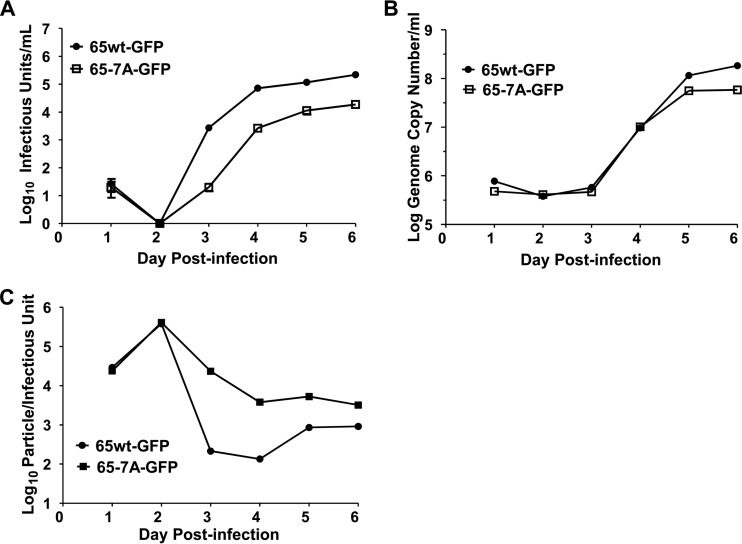
Expression of Grasp65-7A–GFP inhibits assembly of infectious HCMV virus particles. HF cells expressing either Grasp65wt-GFP or Grasp65-7A–GFP were plated and grown to confluence prior to infection with HCMV AD 169 at an MOI of 1. Viral supernatant was collected every 24 h and analyzed for the production of viral progeny in a multicycle virus yield assay. (A) Expression of Grasp65-7A reduces virus yield compared to the yield when Grasp65wt is expressed. At the times indicated, supernatants were collected from HCMV-infected cultures expressing Grasp65wt or mutant Grasp65-7A protein, and infectious virus production was determined as described in Materials and Methods. (B) The release of virus particles containing DNA was not significantly altered by the expression of mutant Grasp65-7A. Quantification of HCMV DNA released into supernatant shows that release of viral DNA is similar in transduced cells. (C) Expression of Grasp65-7A increases the proportion of defective viral particles compared to the proportion in cultures expressing Grasp65wt. The particle/infectious unit ratio was calculated using the data shown in panels A and B. Results are expressed as mean values ± standard deviations.

The AC has been proposed to function as the site of secondary envelopment and virion assembly (18, 20, 21). Reorganization of the Golgi apparatus during the morphogenesis of the AC could contribute to the function(s) of the AC in virion assembly by targeting fully processed glycoproteins to the AC for virion envelopment. Alternatively, reorganization of the Golgi apparatus could optimize interactions between viral envelope proteins and tegument proteins that utilize cytoskeleton-dependent trafficking itineraries to reach the AC (25). Thus, a failure to efficiently fragment and reorganize the Golgi apparatus into the AC in cells expressing the Grasp65-7A mutant protein could result in decreased infectious virus production. Consistent with this possibility, we observed 1- to 2-log reductions in infectious virus in supernatants collected from cell cultures expressing the Grasp65-7A protein compared to the amounts of infectious virus from cell cultures expressing Grasp65wt between days 3 and 6 p.i. in a multicycle virus yield assay (Fig. 10A). These results provided further evidence that the reorganization of the Golgi membranes was required for the efficient production and/or release of infectious particles.

To distinguish whether the decrease in yield of infectious virus particles was secondary to a reduction in the assembly of infectious virions or a deficit in the release of the particles from the cells, we quantified the number of HCMV genomes released into the cell culture supernatant and compared these results to the infectivity of the supernatant. Our results indicated that the numbers of genome copies of viral DNA were only marginally different on days 2 to 4 p.i. in supernatants collected from Grasp65wt- and Grasp65-7A-transduced cell cultures (Fig. 10B). It was also noted that the particle/infectivity ratio of supernatant virus from Grasp-65–7A-transduced cultures was increased compared to the ratio for supernatants from cells transduced with Grasp65wt (Fig. 10C).

**FIG 11:**
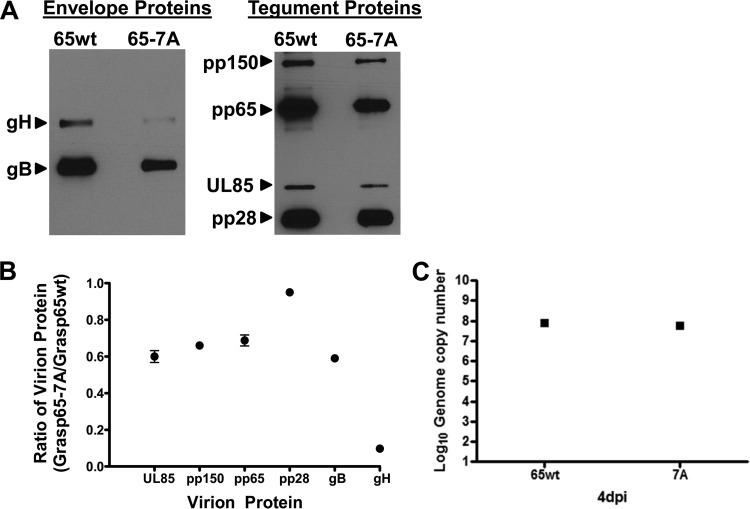
Expression of Grasp65-7A–GFP alters the envelope protein composition of extracellular virions. Transduced cells expressing either Grasp65wt-GFP or Grasp65-7A–GFP were plated and allowed to grow to confluence prior to infection with HCMV AD 169 at an MOI of 1. Extracellular virions were pelleted on day 4 p.i. by high-speed centrifugation through a 25% sorbitol cushion and analyzed by immunoblotting. (A) Expression of mutant Grasp65-7A alters virion composition. Panels show representative blots of envelope proteins (gB and gH), tegument proteins (pp150, pp65, and pp28), and minor capsid protein (pUL85) in extracellular particles collected from cells expressing Grasp65wt or Grasp65-7A. (B) Expression of Grasp65-7A leads to reduced incorporation of gH into extracellular particles compared to that in control samples. Plots show the densitometric ratio of the respective protein bands from Western blots of virions pelleted from infected cells expressing Grasp65-7A or Grasp65wt. Densitometry of the respective bands was accomplished by generating multiple exposures to create a linear range of densities and calculating density using Image Studio Lite version 5.2 software (Li-Cor). Ratios are expressed as mean values ± standard deviations. (C) The genome copy numbers were similar for pelleted virions prepared from supernatants of infected cell cultures expressing Grasp65wt (8.67_log_) and Grasp-7A (8.53_log_).

Because the reduction in the yield of infectious virus from infected cells expressing Grasp65-7A appeared to be due to reduced infectivity of the individual particles, we determined whether the expression of the Grasp65-7A mutant in infected cells altered the incorporation of essential proteins during virion assembly. Viral particles purified from the supernatants of cells transduced with either the Grasp65wt or Grasp65-7A lentivirus and infected with HCMV were analyzed by immunoblotting. Compared to virions isolated from Grasp65wt lentivirus-transduced cultures, viral particles prepared from the supernatant of Grasp65-7A-expressing cells contained approximately 60% of the amounts of the minor capsid protein (UL85), the tegument proteins pp150 (UL32), and pp65, and the envelope glycoprotein gB (Fig. 11A and B). The amount of pp28 (UL99) incorporated into virions produced in from Grasp65-7A expressing cells was nearly equivalent to the level of pp28 detected in virions produced by Grasp65wt expressing cells (Fig. 11A and B). In contrast, we detected an approximately 90% reduction in the amount of the viral envelope glycoprotein gH (UL75) incorporated into virions produced by HCMV-infected, Grasp65-7A-expressing cells compared to the amount in virions produced by Grasp65wt-expressing infected cells (Fig. 11B). Similar quantities of DNA-containing particles were present in equal amounts of virion preparation from infected cells expressing Grasp65wt (8.67_log_) or Grasp-7A (8.53_log_) (Fig. 11C). Importantly, we did not detect a change in the cleavage of the gB precursor and/or migration of either envelope glycoprotein in virions produced in Grasp65wt- or Grasp65-7A-transduced cells, suggesting that Grasp65-7A expression did not result in major alterations in the posttranslational modifications of these envelope proteins. These data suggested that reorganization of the Golgi membrane during morphogenesis of the AC was associated with efficient incorporation of the essential glycoprotein gH into the virion.

## DISCUSSION

In this study, we characterized the effects of HCMV infection and replication on Golgi membrane morphology and investigated the role of Golgi membrane reorganization in AC morphogenesis and the production of infectious virus. A number of reports have previously shown that viruses and intracellular bacteria remodel cellular organelles to create platforms for replication and/or assembly (52–61; reviewed in references 52 to 54). The variety of strategies used by intracellular pathogens to alter the morphology and the lipid and protein composition of cellular organelles likely reflects microbial diversity and distinct requirements for assembling infectious progeny that developed during the adaptation of the pathogens to specific hosts. While some pathogens divert membrane traffic within the endocytic and retrograde pathways, others regulate anterograde/exocytic traffic to support their replication (62, 63). A well-studied example of a microbe driving such a reorganization of intracellular organelles is *Chlamydia trachomatis*, which generates a replication vacuole, the inclusion body, by extensive remodeling of the Golgi apparatus (55, 64). This bacterium encodes a protease, chlamydial protease-like activity factor (CPAF), which cleaves the *cis-*Golgi-resident protein golgin-84, leading to fragmentation of the Golgi apparatus (55). The fragmentation of the Golgi membrane ribbon during chlamydial infection is also dependent on the Rab GTPases Rab6, Rab11, and Rab14 and is thought to be required for the expansion of the replication compartment and bacterial growth (56, 65). Golgi membrane fragmentation coincides with increased lipid acquisition in the inclusion body, and it is postulated that the disruption of Golgi apparatus morphology and function enhances lipid transport to the replication compartment (55). Subsequent studies demonstrated that the accumulation of cholesterol and sphingolipids at the inclusion body requires the activity of multiple SNAREs, including syntaxins 6 and 10, GS15, and VAMP4 (66–69). In addition, chlamydial infection targets the cytoskeleton to stimulate *C. trachomatis* inclusion body morphogenesis (57). Infection leads to the reorganization and modification of microtubules around the vacuole, which is important for the redistribution of Golgi membrane ministacks generated by CPAF-mediated golgin-84 cleavage around the structure (57).

Similar to intracellular bacteria, viruses have been shown to utilize a variety of mechanisms to reorganize cellular membrane transport pathways to generate membrane compartments for replication. Some viruses, such as those in the Picornaviridae and Flaviviridae families, have been shown to hijack cellular enzymes involved in phosphoinositide metabolism to alter membrane lipid composition and remodel the cellular secretory compartment at sites of virus replication (70; reviewed in references 54, 71, and 72). As an example, coxsackievirus B3 (CVB3) recruits the small Ras family GTPase Arf1 and its guanine exchange factor (GEF) GBF1 to membranes via interactions with the CVB3 3A protein, a component of the viral RNA replication complex (70, 73). In addition, CVB3 3A has been shown to recruit phosphatidylinositol-4-kinase IIIβ (PI4KIIIβ) to replication organelles located at ER exit sites independently of GBF1 and Arf1 (74). The recruitment of PI4KIIIβ by the 3A proteins of CVB3 catalyzes the accumulation of phosphatidylinositol-4-phosphate in these membranes, thereby facilitating the binding of the viral replication complex protein 3D^pol^, which is necessary for efficient replication (70; reviewed in reference 71). Of interest is the recent report describing a role of the class II phosphatidylinositol 3-kinase PI3K-C2 in the assembly of infectious HCMV (75). Although the role of this cellular kinase in the cytoplasmic assembly of HCMV was not described, small interfering RNA (siRNA) knockdown of this protein in HCMV-infected cells resulted in a log reduction in virus yield that was associated with a potential defect in virus release from the infected cell (75). In addition to the observations described in this report, other viruses have been shown to induce fragmentation of the Golgi membranes to facilitate their assembly, although the detailed mechanisms used in the modification of this organelle remain undefined (76, 77). For example, human rhinovirus 16 (HRV16) has been reported to cause fragmentation of the Golgi membranes, and HRV16 3A and 3AB proteins were reported to be sufficient for this effect (58); however, whether these proteins alter intra-Golgi membrane vesicle traffic or specifically target proteins that anchor Golgi membrane stacks together has not been determined.

We have determined that the *cis-*Golgi-integral membrane protein Grasp65 was phosphorylated during HCMV infection and that Grasp65 phosphorylation was required for Golgi membrane fragmentation and AC morphogenesis. It should be noted that Golgi membrane fragmentation is not unique to HCMV and has also been described in cells infected with herpes simplex virus 1 (HSV-1); however, our studies have identified a specific Golgi membrane protein that is modified during HCMV infection and has been shown previously to be essential for the maintenance of the Golgi membrane architecture (35, 42, 78). Grasp proteins have been shown to maintain Golgi membrane structure through interactions with golgins, a family of coiled-coiled proteins that serve to tether vesicles to Golgi membranes and that also function to maintain Golgi apparatus morphology (33, 79; reviewed in reference 80). As noted above, golgin-84, which has a critical role in the maintenance of the Golgi membrane ribbon, is cleaved during chlamydial infection, leading to the formation of ministacks that are arranged around the inclusion body; thus, by modifying Golgi membrane proteins important for stacking cisternae, HCMV and chlamydia appear to employ similar strategies for remodeling the organelle.

In our studies, we noted that the fragmentation of the Golgi membranes coincided with the accumulation of the viral tegument protein pp65 in the cytoplasm, suggesting that the localization of pp65 to the cytoplasm could be used as a marker of cells in which viral assembly had progressed to the cytoplasmic phase. Therefore, using the expression of pp65 in the cytoplasm to identify infected cells that were at similar stages of the viral lytic cycle, we demonstrated that Grasp65 was phosphorylated beginning at 48 to 72 h p.i., a time period that coincided with the fragmentation of the Golgi membranes. Because previous studies had clearly demonstrated that phosphorylation of Grasp65 was required for Golgi membrane fragmentation during mitosis, our findings that Grasp65 was phosphorylated provided insight into the mechanism that drives remodeling of the Golgi apparatus during HCMV infection (37, 43, 44, 48, 51). The expression of a Grasp65 mutant, Grasp65-7A, in which putative mitotic kinase phosphorylation sites were mutated to alanines (phosphorylation negative), reduced the efficiency of Golgi membrane fragmentation and AC formation, indicating that the disassembly of the Golgi membrane ribbon was required for AC morphogenesis. These data also suggested the possibility that the activation of mitotic kinases during HCMV infection resulted in modification of Grasp65 and thereby regulated changes in Golgi membrane structure that promoted secondary envelopment. In support of this potential mechanism, it has been reported that Cdk activity late in HCMV infection was required for particle assembly (47). Treatment of HCMV strain Towne-infected cells with the pan-Cdk inhibitor roscovitine, which inhibits Cdk1, Cdk2, Cdk5, Cdk7, and Cdk9, decreased the virus yields by 1 to 2 log but resulted in smaller reductions in the amounts of viral DNA present in the supernatant of infected cells (13). These data were consistent with a role for Cdk activity in the assembly of infectious virus particles. Similar to these findings, the expression of the Grasp65-7A mutant resulted in 1- to 2-log decreases in the production of infectious virus particles on days 2 to 5 p.i. relative to the production of infectious virus in cells transfected with Grasp65wt, as quantified in a multicycle virus yield assay. However, the release of HCMV DNA into the culture supernatant from Grasp65-7A-expressing cells was not decreased significantly compared to the release of HCMV DNA into the supernatants from Grasp65wt-expressing cells, suggesting that the decrease in the yield of infectious virions was likely due to reduced infectivity of the particles released into the supernatant and not secondary to a decrease in the production or release of particles. From these results, we propose that phosphorylation of Grasp65 during HCMV infection, likely by CDK1 and/or, potentially, by PLK1 or ERKs, reduced the capacity of this resident Golgi membrane protein to tether the Golgi cisternae, thus allowing the Golgi membranes to fragment during AC morphogenesis.

While HCMV infection initially induced Golgi membrane fragmentation, Golgi apparatus-derived membranes eventually coalesced to form a ringlike structure that defined the outer boundary of the AC. We could speculate that, following the initial Golgi membrane fragmentation, subsequent dephosphorylation of Grasp65 could aid in the process of AC formation; however, the removal of phosphate groups from Grasp65 was not observed by immunoblot analyses of lysates from HCMV-infected cells. It is more likely that the reorganization of Golgi membranes into the ring forming the outer boundary of the AC is driven by interactions with the cytoskeleton. It is known that Golgi membrane positioning depends on a balance between plus- and minus-end-directed microtubule motors (reviewed in reference 81). Because the AC overlaps the MTOC, it is possible that microtubule motor dynamics control the morphology of the Golgi apparatus and formation of the AC during infection (82). Interference with minus-end microtubule motor activity has been shown to disrupt the morphology of the AC and reduce the production of infectious virus (83). In addition, we have previously shown that the HCMV tegument protein pp150 interacts with the microtubule motor-associated protein bicaudal D1, and although the consequences of this interaction remain incompletely defined, inhibition of this interaction decreased the assembly of infectious virus (25). In uninfected cells, bicaudal D1 interacts with Rab6, a Golgi membrane-associated GTPase that regulates Golgi membrane-ER transport and maintains Golgi membrane morphology through its interactions with golgins (for a review, see reference 84). Thus, it is likely that the interactions between virion proteins, microtubule motors, and the membrane transport machinery all contribute to AC morphogenesis. As previously noted, several proteins have been shown to play a role in the disruption of Golgi membrane structure and the formation of the inclusion body during chlamydial infection (56). Thus, HCMV could utilize a strategy similar to that of chlamydia in that the virus could manipulate multiple intracellular pathways simultaneously during the morphogenesis of the AC. The phosphorylation of key components involved in membrane transport by mitotic kinases could serve to fine-tune these activities (37, 47).

Finally, the data acquired using the Grasp65-7A mutant indicated that the reorganization of the Golgi apparatus was part of the AC morphogenesis and required for optimal incorporation of the viral glycoproteins into the infectious particle. We have previously demonstrated that reduced amounts of gH in the envelope of HCMV were associated with reduced infectivity, suggesting that virions produced from cells expressing the Grasp65-7A mutant could be less infectious secondary to the decreased amounts of this essential glycoprotein in the virion envelope (85). Based on the model of chlamydial inclusion body morphogenesis, we hypothesize that reorganization of the Golgi apparatus during HCMV infection may be required to ensure the efficient transport of membranes containing virion proteins into the AC.

In summary, in this report, we have provided evidence that Grasp65 and mitotic kinases are key regulators in the reorganization of intracellular membranes required for the morphogenesis of the AC and, subsequently, for the production of infectious particles.

## MATERIALS AND METHODS

### Cells and viruses.

Human cytomegalovirus strain AD 169 was propagated in human fibroblast (HF) cells grown in Dulbecco modified Eagle medium (DMEM) supplemented with 5% fetal bovine serum (FBS) and penicillin-streptomycin. Cidofovir was provided in medium by Mark Prichard (Department of Pediatrics, University of Alabama—Birmingham, Birmingham, AL). Virus stocks were prepared by collecting supernatants and cells from cultures exhibiting 100% cytopathic effect, clarified to remove cells and debris, and stored at −80°C until utilized. For infections, HF cells were plated, allowed to grow to confluence, and then incubated for an additional 3 days prior to serum starvation for 72 h. Cells were infected with HCMV strain AD 169 at a multiplicity of infection (MOI) of 1. Lentiviruses were prepared in 293T cells using the ViraPower lentiviral packaging mix (Thermo Fisher, Waltham, MA), except that Polyfect (Qiagen, Valencia, CA) was used as the transduction reagent.

### Antibodies.

The mouse monoclonal antibodies (MAbs) against viral proteins utilized in this project included antibodies to pp150 (36-14), pp28 (41-18), pp65 (28-19), IE1 (p63-27), gB (27-156), gM (IMP), UL85 (pUL85), UL44 (28-21), and gH (AP86). The commercially available antibodies used in these studies were anti-Grasp65 rabbit polyclonal antibody (Thermo-Fisher, Waltham, MA), anti-GM130 rabbit polyclonal antibody (Thermo-Fisher, Waltham, MA), anti-GM130 MAb (BD Biosciences, San Jose, CA), anti-golgin-245 MAb (BD Biosciences, San Jose, CA), anti-actin MAb (EMD Millipore, Billerica, MA), anti-GFP antibody (BD Biosciences, San Jose, CA), and anti-HCMV UL57 antibody (Virusys, Taneytown, MD).

### Virus infectivity and qPCR.

To determine viral titers, fibroblasts were infected with serial dilutions of supernatants and incubated for 24 h. After fixation in ethanol, cells were stained with antibody directed against IE1-72 (p63-27). The number of IE1^+^ cells was counted, using a fluorescence microscope, at the lowest dilution that gave at least 100 IE1^+^ cells/well to determine the number of infectious units/ml.

The genome copy number was determined by quantitative PCR (qPCR) using primers 5′-GAG CCC GAC TTT ACC ATC CA-3′ and 5′-CAG CCG GCG GTA TCG A-3′ with the probe 5′-VIC-ACC GCA ACA AGA TT-MGB-NFQ-3′ (MGB-NFQ, minor groove binder-nonfluorescent quencher) (86) and, using plasmid pMP582, normalized to WHO international standards to provide absolute quantification.

### Plasmids and constructs.

Grasp65 cDNA was purchased from Genecopeia (Rockville, MD) and cloned into the pEGFP-N1 expression vector (TaKaRa Bio USA, Mountain View, CA). The mitotic phosphorylation-negative pEGFP-N1-Grasp65-7A construct was generated using overlapping PCR with the following primer pairs: S189A-F, 5′-ACTG GGG AGA GGG CGC CCT GGG ATG T-3′, and S189A-R, 5′-ACTG ACA TCC CAG GGC GCC CTC TCC-3′; S274A-S276A-S278A-F, 5′-GGG GCT CCC GCC CAC GCT GCT CCA GAC CCT GAT GGA C-3′, and S274A-S276A-S278A-R, 5′-GTC CAT CAG GGT CTG GAG CAG CGT GGG CGG GAG CCC C-3′; T216A-F, 5′-CCT GGC GCC CCA CCA CCT TCT G-3′, and T216A-R, 5′-CAG AAG GTG GTG GGG CGC CAG G-3′; S373A-F, 5′-CTG GAC GCC CCA GGT GCC CAA GCC-3′, and S373A-R, 5′-GGC TTG GGC ACC TGG GGC GTC CAG-3′; and S397A-F, 5′-GCA GCC GCA CCA GAA GAT GGG-3′, and S397A-R, 5′-CCC ATC TTC TGG TGC GGC TGC-3′. Cloning into pEGFP-N1 was done using the HindIII (F) and Sac2 (R) sites. The insert was generated using the overlapping PCR products with the following pEGFP-N1 primers: forward, 5′-ACTG AAG CTT ATG GGC CTG GGC GTC AGC GCT GAG-3′, and reverse, 5′-ACTGT GGT AGA GAT CTG GGC CTG-3′. Gene sequences were amplified by PCR and inserted into the pLVX-Puro vector to generate recombinant lentiviruses expressing wt or mutant Grasp65-EGFP fusions. The primers used for the generation of lentiviruses were as follows: forward, 5′-ATG CGA ATT CAT GGG CCT GGG CGT C-3′, and reverse, 5′-ATG CTC TAG ATT ACT TGT ACA GCT CGT CCA TGC CGA G-3′. Isolation of plasmid DNA was done using the endotoxin-free midiprep kit (Thermo-Fisher, Waltham, MA).

### SDS-PAGE and immunoblotting.

Sodium dodecyl sulfate-polyacrylamide gel electrophoresis (SDS-PAGE) was conducted under reducing conditions, followed by transfer of the gel to nitrocellulose paper for immunoblotting. Detection of target proteins with primary antibodies was followed by incubation with horseradish peroxidase (HRP)-conjugated secondary antibodies and then chemiluminescence detection. For comparison of the protein contents of purified extracellular particles, multiple exposures of Western blots were generated and scanned to create digital images to be analyzed using Image Studio lite version 5.2 software (Li-Cor, Lincoln, NE). The signal densities were determined for at least 4 different exposures of each blotted protein and plotted to define a densitometry curve. Values from the linear portion of the densitometry curve were used to calculate the density ratios comparing protein quantities in Grasp65-7A versus Grasp65wt extracellular particles. A graph in which the results were expressed as the mean ± SD was generated with GraphPad 5.0 software.

### Grasp65 phosphatase assays.

HF cells were plated in 60-mm dishes, allowed to grow to confluence, and incubated for 3 days prior to serum starvation for 24 h. Cells were infected with HCMV AD 169 at an MOI of 1. A confluent cell population and an asynchronous cell population were utilized as negative and positive controls, respectively, for Grasp phosphorylation. For the asynchronous cell population, cells were plated at a subconfluent density and, 24 h later, were serum starved for 24 h. Medium containing serum was then added, and cells were returned to the incubator for 22 h prior to collection. Cells were lysed in HEPES lysis buffer containing 0.5% NP-40 on ice, and the lysate was incubated with a protease inhibitor cocktail (Research Products International, Prospect, IL) and/or a phosphatase inhibitor cocktail (PIC; RPI, Prospect, IL). Cell lysates were incubated with calf intestinal phosphatase (New England Biolabs, Ipswich, MA) at a concentration of 10,000 U/µg lysate in NEB buffer 3 at 37°C for 3 h to remove phosphates from protein. SDS loading buffer (5×) was added to the lysate prior to boiling and loading on gels. Equivalent protein loading was quantified using the Pierce bicinchoninic acid (BCA) protein assay kit (Thermo Scientific, Waltham, MA).

### Confocal microscopy.

HF cells were grown on glass coverslips within 24-well plates and fixed in 4% paraformaldehyde (PFA) in phosphate-buffered saline (PBS). Cells were permeabilized with 0.1% Triton X-100 in PBS prior to blocking with 5% normal goat serum in PBS. Coverslips were then incubated with monoclonal antibodies or polyclonal primary antibodies in PBS containing 10% normal goat serum. When using rabbit polyclonal antibodies, the ChromPure human IgG Fc fragment (Jackson ImmunoResearch, West Grove, PA) was included with all primary and secondary antibody incubations. After incubation with primary antibodies, coverslips were washed in PBS containing 0.1% Tween 20. Alexa Fluor secondary antibodies were used in this study, including Alexa Fluor 488 (green), Alexa Fluor 594 (red), and Alexa Fluor 647 (blue) (Life Technologies, Carlsbad, CA). Nuclei were identified with 4′,6-diamidino-2-phenylindole (DAPI) staining. Coverslips were mounted with ProLong gold antifade solution (Cell Signaling Technology, Danvers, MA). The Golgi membrane length was measured using the Trace feature in FluoView software (Olympus Corporation), and the results are detailed in Fig. S1 in the supplemental material.

## SUPPLEMENTAL MATERIAL

Figure S1  Measurement of morphometry of transverse length of Golgi membranes and lengths of Golgi membrane fragments. (A) Day 1 infected cells stained with anti-pp65 (blue), anti-Grasp65 (red), and anti-GM130 (green) antibodies. Merged channel yields yellow of overlapping signals. (B) Green line demarcates Golgi membranes from panel A, and analytic software provided quantification of lengths. (C) Fragmented Golgi membranes on day 3 postinfection stained with anti-pp65 (blue), anti-Grasp65 (red), and anti-GM130 (green) antibodies. Continuous Golgi membrane fragments are numbered. (D) Fragment lengths are demarcated by green lines, and quantification of fragment lengths was determined by software. Download Figure S1, PDF file, 0.1 MB
