# Supplementary material for: Phosphorylation of Golgi Peripheral Membrane Protein Grasp65 Is an Integral Step in the Formation of the Human Cytomegalovirus Cytoplasmic Assembly Compartment
Source: mBio. 2016 Oct 4;7(5):e01554-16. doi: 10.1128/mBio.01554-16 (PMC5050342; doi:10.1128/mBio.01554-16)
Supplement: Figure S1 — Measurement of morphometry of transverse length of Golgi membranes and lengths of Golgi membrane fragments. (A) Day 1 infected cells stained with anti-pp65 (blue), anti-Grasp65 (red), and anti-GM130 (green) antibodies. Merged channel yields yellow of overlapping signals. (B) Green line demarcates Golgi membranes from panel A, and analytic software provided quantification of lengths. (C) Fragmented Golgi membranes on day 3 postinfection stained with anti-pp65 (blue), anti-Grasp65 (red), and anti-GM130 (green) antibodies. Continuous Golgi membrane fragments are numbered. (D) Fragment lengths are demarcated by green lines, and quantification of fragment lengths was determined by software. Download [file mbo005163004sf1.pdf]

Supplemental Material: **Rebmann, et.al.** **Phosphorylation of the Golgi peripheral membrane protein Grasp65 is an integral step in formation of the human cytomegalovirus cytoplasmic assembly compartment.**

Rebmann Suppl Fig 1

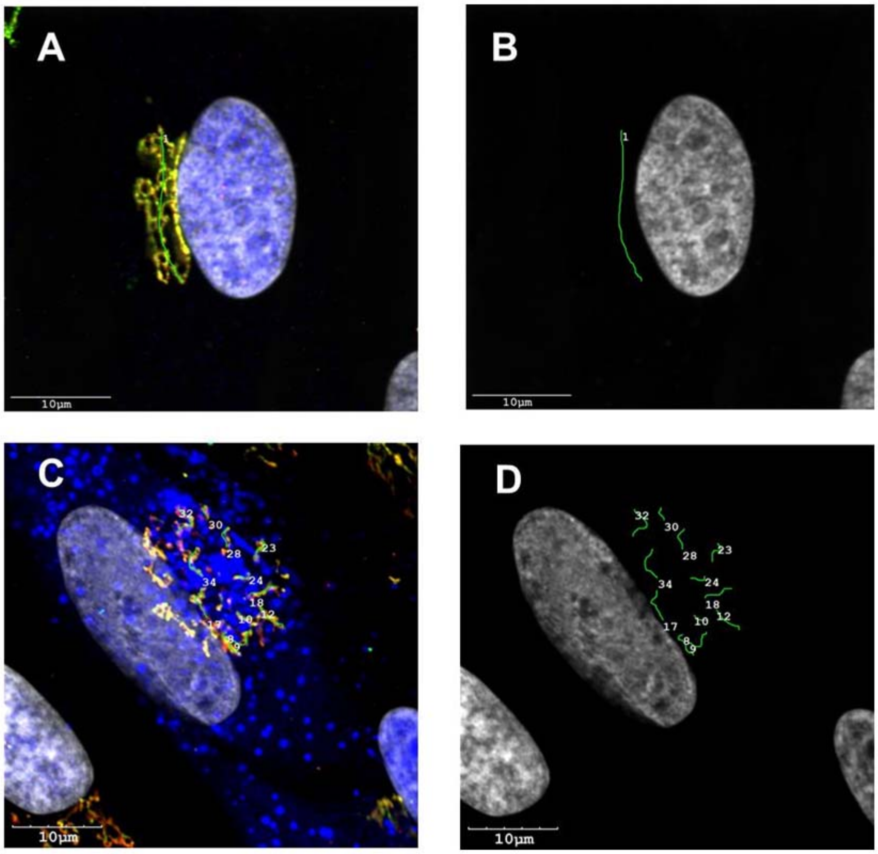

Supplemental Figure 1. Measurement of morphometry of transverse length of Golgi and lengths of Golgi fragments. (A) Day 1 infected cells stained with anti-pp65 (blue), anti-Grasp65 (red), and anti-GM130 (green). Merged channel yields yellow of overlapping signals. **(B)** Green line demarcates Golgi from Panel A and analytics of software provided quantification of length. **(C)** Fragmented Golgi on day 3 post-infection stained with anti-pp65 (blue), anti-Grasp65 (red), and anti-GM130 (green). Continuous Golgi fragments are numbered**. (D)** Fragment lengths demarcated by green lines and quantification of fragment length determined by software.
